# Comparison of nonsense-mediated mRNA decay efficiency in various murine tissues

**DOI:** 10.1186/1471-2156-9-83

**Published:** 2008-12-05

**Authors:** Almoutassem B Zetoune, Sandra Fontanière, Delphine Magnin, Olga Anczuków, Monique Buisson, Chang X Zhang, Sylvie Mazoyer

**Affiliations:** 1 Laboratoire de Génétique Moléculaire, Signalisation et Cancer UMR5201 CNRS, «Equipe Labellisée par la Ligue Nationale contre le Cancer», Université Lyon 1, Université de Lyon, Faculté de Médecine, 8 avenue Rockefeller, 69373 Lyon cedex 08, France; 2 Unité de Prévention et d'Epidémiologie Génétique UMR5558 CNRS, Université Lyon 1, Université de Lyon, Centre Léon Bérard, 28 rue Laënnec, 69378 Lyon cedex 08, France

## Abstract

**Background:**

The Nonsense-Mediated mRNA Decay (NMD) pathway detects and degrades mRNAs containing premature termination codons, thereby preventing the accumulation of potentially detrimental truncated proteins. Intertissue variation in the efficiency of this mechanism has been suggested, which could have important implications for the understanding of genotype-phenotype correlations in various genetic disorders. However, compelling evidence in favour of this hypothesis is lacking. Here, we have explored this question by measuring the ratio of mutant versus wild-type *Men1 *transcripts in thirteen tissues from mice carrying a heterozygous truncating mutation in the ubiquitously expressed *Men1 *gene.

**Results:**

Significant differences were found between two groups of tissues. The first group, which includes testis, ovary, brain and heart, displays a strong decrease of the nonsense transcript (average ratio of 18% of mutant versus wild-type *Men1 *transcripts, identical to the value measured in murine embryonic fibroblasts). The second group, comprising lung, intestine and thymus, shows much less pronounced NMD (average ratio of 35%). Importantly, the extent of degradation by NMD does not correlate with the expression level of eleven genes encoding proteins involved in NMD or with the expression level of the *Men1 *gene.

**Conclusion:**

Mouse models are an attractive option to evaluate the efficiency of NMD in multiple mammalian tissues and organs, given that it is much easier to obtain these from a mouse than from a single individual carrying a germline truncating mutation. In this study, we have uncovered in the thirteen different murine tissues that we examined up to a two-fold difference in NMD efficiency.

## Background

Messenger RNA (mRNA) quality control is essential to assure the fidelity of gene expression. It is achieved through several mechanisms among which Nonsense-Mediated mRNA Decay (NMD) plays a major role. The NMD pathway detects and degrades mRNAs containing premature termination codons (PTCs), thereby preventing the accumulation of potentially harmful truncated proteins [1]. NMD is evolutionary conserved, although the way PTCs are recognized by the surveillance machinery may differ between species. In mammals, PTC recognition depends upon the position of the stop codon within the transcript. The normal stop codon is nearly always located in the last exon [2], so any stop codon located >50 nucleotides upstream of the last exon-exon boundary will be considered as premature. Recognition is thought to occur during a pioneering round of translation, and exon-exon boundaries are delimited on the transcript by exon-exon junction complexes (EJCs) deposited during splicing [3].

The NMD pathway has important repercussions on the manifestation of human genetic diseases. Although truncating mutations, i.e. mutations that introduce a PTC in the coding sequence, are usually not expected to lead to substantial amounts of truncated proteins, there are situations where the mutant transcripts escape NMD. This happens when the PTC is located in the last exon [4,5], at the beginning of the coding sequence where it can possibly induce translation reinitiation [6-8], or when the PTC is absent from the mutant transcript because of exon skipping [4,9]. Differences in the ability of PTC-containing mRNAs to trigger NMD have been found to explain several phenotype/genotype correlations [10,11]. The most striking examples are β-thalassemia (MIM 141900), which is commonly a recessive disorder, but shows a dominant inheritance pattern when mutations in the *β*-*globin *gene are NMD-incompetent [12], and two different peripheral neuropathies that are both the result of truncating mutations in the *SOX10 *gene that differ in their ability to trigger NMD [13].

NMD has also long been suspected to influence the clinical outcome of genetic diseases as a result of intertissue variation in the efficiency of this mechanism. Variation in NMD efficiency could lead to the expression of variable amounts of truncated proteins depending on the tissue considered, which might in turn help to explain why truncating mutations in ubiquitously or widely expressed genes appear to affect only a very specific and small subset of tissues in humans. Evidence for variation of NMD efficiency comes from the observation that in patients carrying PTCs in the collagen X gene, low abundance of mutant transcripts was observed in cartilage cells due to NMD, while in lymphoblasts and bone cells, no NMD-mediated mRNA diminution was observed [14].

Here, we have explored the question of variation in NMD efficiency by measuring the relative amount of a PTC-containing *Men1 *transcript, as compared to the transcript level of the wild-type allele, in 13 tissues taken from heterozygous *Men1 *knockout mice. We observed that there is up to a two-fold difference in the efficiency of NMD between the examined tissues.

## Results

### NMD degradation of mutant Men1 transcripts in *Men1*^+/Δ ^mice model

**Figure 1:**
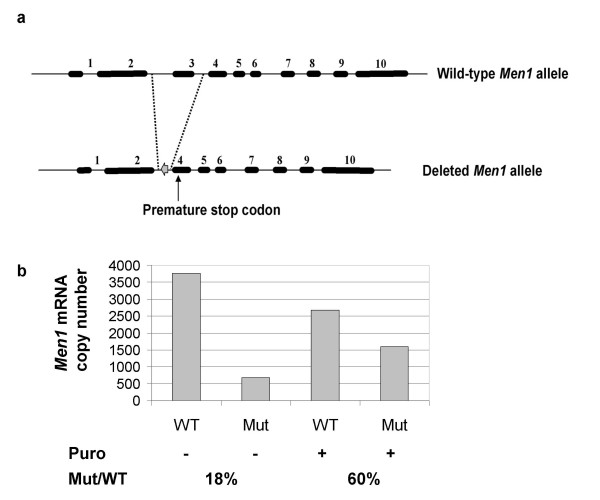
**Description of the *Men1 *mouse model used in the analysis**. (a) Schematic representation of the mutant and of the wild-type allele of the *Men1 *gene in the heterozygous *Men1 *mutant mice. Black boxes and lines represent exons and introns respectively. The grey horizontal arrow depicts a *loxP *sequence, and the black vertical arrow shows the location of the premature stop codon generated by exon 3 deletion. The strategy used to delete *Men1 *exon 3 in mice has been described previously [17]. (b) Transcript copy number were measured by quantitative RT-PCR using 50 pg of RNA extracted from *Men1*^+/Δ ^mouse embryonic fibroblasts (MEF) untreated or treated with puromycin (Puro). Specific amplification of either the wild-type (WT) or the mutant (Mu) transcript lacking exon 3 was achieved using the primers described in the Methods section.

The murine *Men1 *gene, like its human ortholog, contains 10 exons, of which nine are coding. It has been described that four different *Men1 *transcripts displaying the same coding sequence are produced through alternative splicing of intron 1 [15,16]. The mouse model we have used in this analysis has been generated by deleting exon 3 in one allele of *Men1 *[17]. This deletion introduces a PTC in exon 4 of the coding sequence, 164 ter (Figure 1a). Heterozygous *Men1 *knockout (*Men1*^+/Δ^) mice of both sexes develop normally and are fertile and healthy until they are at least 7 months of age [17]. *Men1*^+/Δ ^mouse embryonic fibroblasts (MEF) were used to verify that PTC-containing *Men1 *transcripts were subjected to NMD as expected, given the presence of six exon-exon junctions downstream of the PTC. We found by real time quantitative RT-PCR that mutant transcripts were present at levels of 18% and 60% of their wild-type counterpart without and with the NMD inhibitor puromycin, respectively (Figure 1b).

### Relative amount of *Men1 *mutant transcripts

**Figure 2:**
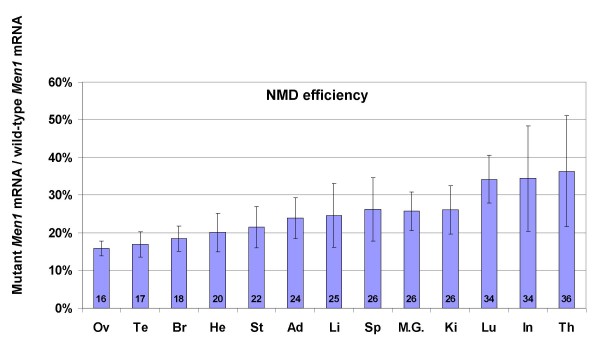
**NMD efficiency in 13 tissues of mice heterozygous for a truncating mutation in the *Men1 *gene**. NMD efficiency is expressed as a ratio of the amount of mutant *Men1 *transcripts versus wild-type species, as measured by quantitative RT-PCR. Each bar on the graph represents the mean value of at least 3 independent measurements (± standard deviation) in four samples of the same tissue (two samples only were used for testis and ovary).

We measured the ratio of mutant versus wild-type *Men1 *transcripts in 13 different tissues taken from four *Men1*^+/Δ ^mice. The values ranged from 16% to 36%, as seen on Figure 2. Statistical analysis showed that there was a significant difference between two distinct groups of tissues; one comprised of testis/ovarian, brain and heart (average ratio of 18%), and the other of lung, intestine and thymus (average ratio of 35%).

**Figure 3:**
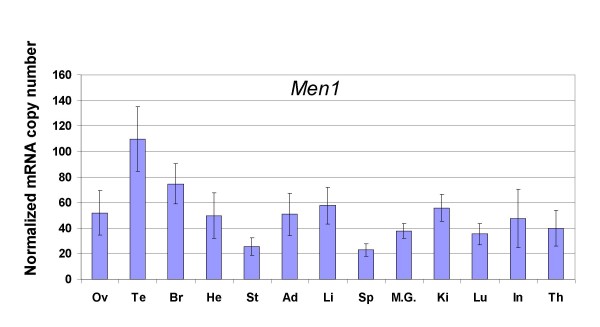
***Men1 *transcript levels in 13 murine tissues**. Transcript levels (copy number per 50 pg of RNA extracted from each of the mentioned tissue) were measured by quantitative RT-PCR, and normalized to the level of *Hprt1 *and *β*-*actin *transcripts. Each bar on the graph represents the mean value of 3 independent measurements (± standard deviation) in four samples of the same tissue (two samples only were used for testis and ovary).

The *Men1 *gene has been reported to be expressed in all the tissues examined, albeit at variable levels, by Northern blot analysis [16,18,19]. Quantification by quantitative RT-PCR analysis confirmed this finding. The number of copies was normalized to the amount of *Hprt1 *and *β*-*actin *transcripts (Figure 3). The lowest level of *Men1 *transcripts was found in spleen and stomach while the highest level was found in testis and brain (nearly 4-fold difference); the differences observed in the other tissues were not statistically significant. No correlation between NMD efficiency and *Men1 *transcript levels was observed.

### Expression levels of the genes involved in NMD

NMD triggering in humans, based on present models, hinges on the interaction of critical protein components, hUpf2, hUpf3a/hUpf3b, that are bound to the EJC (composed in the cytoplasm of Y14, Magoh, eIF4AIII and hBarentsz) with a molecule, hUpf1, recruited to the post-termination complex deposited at the PTC [3]. The SMG1 kinase, in turn, binds to this complex and phosphorylates hUpf1, which leads to subsequent recruitment of RNA degrading enzymes. The association of these proteins with the post-spliced mRNA is essential as their depletion, mediated by RNA interference, has been shown to suppress degradation of NMD substrates [20,21]. Furthermore, NMD can be recapitulated by tethering the hUpf factors, Magoh, Y14, eIF4AIII, or hBarentsz within the 3'UTR of a reporter mRNA in human cells [22].

**Figure 4:**
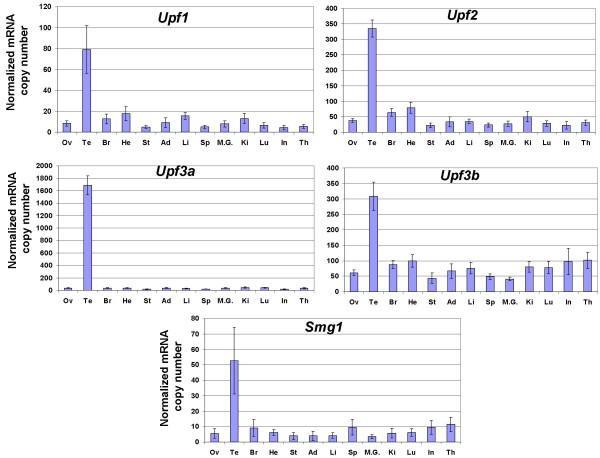
**Transcript levels of the *Upf1-3 *and *Smg1 *genes in 13 murine tissues**. Transcript levels (copy number per 50 pg of RNA extracted from each of the mentioned tissues) were measured by quantitative RT-PCR using the same samples as previously, and normalized to the level of *Hprt1 *and *β*-*actin *transcripts. Each bar on the graph represents the mean value of at least 3 independent measurements (± standard deviation) in four samples of the same tissue (two samples only were used for testis and ovary). Please note that the scale showing the normalized number of transcripts is different for each gene. Ov: ovary; Te: testis; Br: brain; He; heart; St; stomach; Ad: adrenal gland; Li: liver; Sp: spleen; M.G.: mammary gland; Ki: kidney; Lu: lung; In: intestine; Th: thymus.

**Figure 5:**
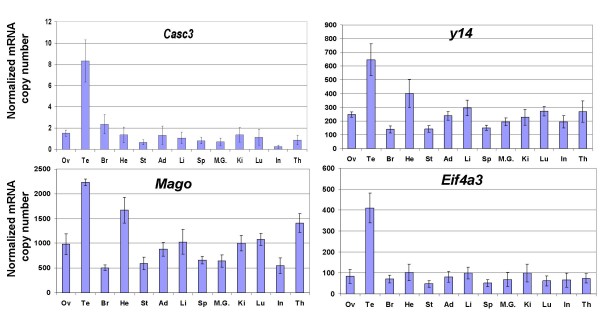
**Transcript levels of the genes encoding the EJC core proteins in 13 murine tissues**. Transcript levels (copy number per 50 pg of RNA extracted from each of the mentioned tissues) were measured by quantitative RT-PCR using the same samples as previously, and normalized to the level of *Hprt1 *and *β*-*actin *transcripts, as in Figure 4.

**Figure 6:**
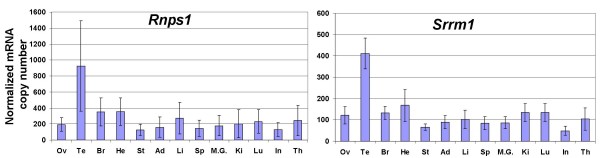
**Transcript levels of the genes encoding two splicing factors involved in the NMD mechanism in 13 murine tissues**. Transcript levels (copy number per 50 pg of RNA extracted from each of the mentioned tissues) were measured by quantitative RT-PCR using the same samples as previously, and normalized to the level of *Hprt1 *and *β*-*actin *transcripts, as in Figure 4.

Human *UPF1 *[23], *UPF2 *[24], *MAGOH *[25], *CASC3 *(encoding hBarentsz) [26], and *EIF4A3 *[27] genes have been shown to be ubiquitously expressed by Northern blot, but their level of expression varies among tissues. In order to more precisely determine the extent of these variations in mice, and to investigate the possibility of an eventual link with NMD efficiency, we have measured the amount of mRNAs transcribed from the genes encoding nine of the proteins involved in NMD by real time quantitative RT-PCR: *Upf1*, *Upf2*, *Upf3a*, *Upf3b*, *Smg1 *(also known as *RICKEN*), *y14 *(also known as *Rbm8a*), *Mago*, *Casc3 *and *Eif4a3 *(Figure 4 and 5). We also analysed the level of expression of the genes encoding the splicing coactivators/alternative splicing factors that have been shown to interact with the EJC, SRm160 and RNPS1 (*Srrm1 *and *Rnps1 *respectively in mice) (Figure 6), given the described link between efficiency of splicing and efficiency of NMD [28], and given the fact that the abundance of RNPS1 has been reported to determine the variability in efficiency of NMD in three strains of HeLa cells [29]. The same RNA samples were used as previously described for measuring levels of *Men1 *transcripts, and the number of copies was likewise normalized to the amount of *Hprt1 *and *β*-*actin *transcripts. A great variation in the mean level of expression of the eleven studied genes was observed: *Mago *transcripts (1000 copy per 50 pg of total RNA) are 500 times more abundant than *Casc3 *transcripts (2 copies). Included within these boundaries are *Rnps1 (*275 copies), *y14 *(265 copies), *Upf3a *(160 copies), *Srrm1 *(130 copies), *Eif4a3 *(100 copies), *Upf3b *(90 copies), *Upf2 *(60 copies), *Upf1 *(15 copies) and *Smg1 *(10 copies).

Concerning tissues variations, no statistical difference in the amount of transcripts was seen in the case of *Rnps1 *(Figure 6). For *y14*, testis and heart were significantly different from ovary, brain, stomach, adrenal, spleen, mammary gland, kidney and intestine (Figure 5). Testis, heart and thymus were significantly different from brain, intestine, mammary gland, spleen and stomach for *Mago *(Figure 5). For the remaining genes, the only tissue showing a significant variation was testis (Figure 4, 5, 6), with an amount of transcripts 4 to 50 times higher than in all the other tissues. No clear correlation could be drawn between the efficiency of NMD and the level of expression of any of the mRNAs encoding proteins essential to this mechanism.

## Discussion

Intertissue variation in NMD efficiency has long been suggested to modulate the clinical outcome of genetic diseases. Indeed, such a variation would mean that in some tissues more than in others, translation of residual mRNAs containing PTCs could potentially lead to functionally important expression of truncated proteins and influence the phenotype.

The first hint that NMD efficiency could vary between tissues from the same individual came from the study by Bateman and colleagues on truncating mutations in the collagen X gene, *COL10A1*, responsible for Schmid metaphyseal chondrodysplasia (MIM 156500) [14]. They showed that NMD completely removed the mutant *COL10A1 *transcripts in cartilage tissue and cells, but not in non-cartilage cells. However, this *COL10A1 *study presented two particularities that could restrict the notion of NMD efficiency variation to this gene. Firstly, nonsense-mediated reduction in abundance of nonsense *COL10A1 *mRNAs is not governed by the same parameters as those in the classical process. Indeed, competency for NMD in this case is specified by the 3'UTR and mRNA decay is only triggered by truncating mutations introducing a PTC in a 3' region of the terminal exon, which contain more than 90% of the coding sequence [30]. Secondly, the *COL10A1 *gene is expressed exclusively in cartilage cells, and therefore, NMD of *COL10A1 *PTC-containing transcripts in non-cartilage cells (bone cells and lymphoblasts) was assessed on "illegitimate" transcripts, i.e. tissue-specific transcripts found at very low levels in non-expressing cells. A correlation between the amount of transcripts and the extent of degradation by NMD was not observed in the case of the *Men1 *gene in our study. However, *Men1 *is ubiquitously expressed and variations of a maximum 5-fold were observed amongst the studied tissues. It remains possible that illegitimate *COL10A1 *transcripts are not translated and that this is the reason why they are not subjected to NMD.

Another related, but distinct matter, concerns interindividual variation in NMD efficiency. Patients carrying identical mutations can show variable reduction of mutant transcript levels in the same cell type [4,31]. In the study of Linde and coll., interindividual variability in NMD efficiency was not only observed in the case of the CFTR transcripts in nasal epithelial cells from patients carrying the W1282X mutation but also in the case of five natural NMD substrates in epithelial cell lines derived from two unrelated patients carrying the same mutations [31]. While intertissue variations of NMD efficiency are probably the result of differences between tissues in the level of expression of the proteins involved in NMD, interindividual variations are likely to be linked to DNA variations in the human genome within the genes encoding the proteins involved in NMD. These two levels of variations are intermingled when the efficiency by which the same truncating mutation triggers NMD is compared in different tissues of different individuals, as in the study by Resta et al [32], and it is therefore not possible in these situations to estimate the contribution of each phenomenon.

Given the difficulties to obtain different tissues from a single patient, mouse models appeared as an attractive alternative to evaluate the efficiency of NMD in multiple mammalian tissues. Indeed, this question was addressed in a study using the *gus*^*mps *^mouse, a model of mucopolysaccharidosis type VII (MIM 253220), which harbours a NMD-competent truncating mutation in the ubiquitously expressed β-glucuronidase gene [24]. However, only homozygous animals were studied, which precluded measurement of steady-state levels of mutant versus wild-type transcripts in different tissues of the same mouse. Their analysis, performed by Northern blot, reported that all eight tissues examined (thymus, spleen, skeletal muscle, liver, kidney, lung, heart and brain) showed efficient degradation of the mutant transcript as it was undetectable in Gus null mutant mice [24], but was not able to uncover discrete variations in NMD efficiency. A recent study performed on a knockin mouse model in which one wild-type *Chek2 *allele has been replaced by an allele carrying the 1100delC mutation showed that the mutant transcripts undergo NMD in MEFs and in four tissues (brain, heart, kidney and liver), but to varying degrees [33]. This analysis was performed by quantitative RT-PCR on RNA extracted from tissues taken from wild-type, *Chek2 *1100delC heterozygous and *Chek2 *1100delC homozygous animals. The RT-PCR protocol used did not allow the discrimination between the wt and the mutant *Chek2 *transcript species and therefore, NMD efficiency was expressed as the amount of both transcripts normalized to *GAPDH *transcripts. Variations were observed, but it couldn't be ruled out that they result from variations in the levels of the *GAPDH *transcripts, as these have been reported to vary depending on the tissues examined [34].

Here, we have quantified for the first time the extent of variation in NMD efficiency by measuring the ratio of mutant versus wild-type *Men1 *transcripts in 13 tissues taken from mice heterozygous for a truncating mutation, and have found up to a two-fold difference. The organs most frequently involved clinically in the Men1 syndrome (the parathyroids, the pituitary and the pancreas) were not all tested in our analysis; furthermore, it has not been possible to verify the expression of a truncated menin protein as all the available antibodies are directed against the C-terminal part of the Menin protein. Therefore, we have not been able to assess whether the differences in NMD efficiency that we have uncovered plays a role in the physiopathology of the disease in the *Men1*^+/Δ ^mice.

Although NMD efficiency varies depending upon the gene, we do not expect that the extent of variation that we have observed is specific to the *Men1 *gene. However, our results need to be confirmed with other mouse models carrying a heterozygous truncating mutation in ubiquitously-expressed genes. Additionally, it would be interesting to also test physiologic NMD substrates. Unfortunately, several features shared by these physiologic substrates limit their use to disclose intertissue variations of NMD efficiency. Indeed, the examination of the expression profile in human cells depleted of hUpf1 revealed that most upregulated transcripts result from alternative splicing events or from the use of alternative translation start site that introduce upstream open reading frames [35]. Both mechanisms show striking variation across tissue types, and therefore, it would not be possible to attribute variations in PTC+ transcript levels in various tissues to differences in NMD efficiency, as they could also be due to differences in the levels of alternative splicing, or in the use of alternative translation start sites.

NMD efficiency is sometimes calculated as the ratio between the amount of mutant transcripts in the absence and in the presence of a NMD inhibitor (often a translation inhibitor) [31,32,36], which would have the advantage in the present case to bypass the aforementioned problem. However, notwithstanding the fact that it is impossible to inhibit NMD in non-growing cells, it is to be noted that the level of up-regulation upon NMD inhibition is highly variable among NMD-sensitive transcripts and it has never been demonstrated that this level correlates with NMD efficiency. On the contrary, the results we obtained when studying *BRCA1/2 *transcripts bearing truncating mutations showed that it is not the case [4,37].

It has been shown recently that RNPS1, a protein component of the EJC, could determine the variability of NMD efficiency in three strains of HeLa cells [29]. However, we did not find in our study on *Men1*^+/Δ ^mice a correlation between NMD efficiency in tissues and the level of transcripts encoding RNPS1, no more than with the levels of the other transcripts that we have tested. It remains possible that these transcript levels are not reflective of the amount of proteins present in these tissues.

## Conclusion

The level of intertissue variations that we have found might be lower than expected based on previous results [14]. Nevertheless, if it were to reflect the situation in humans, there may be circumstances where this two-fold difference is sufficient to modify the ultimate phenotype by allowing the expression of twice as many aberrant truncated proteins with potential dominant-negative or gain-of-function in particular tissues compared to others. Furthermore, the observed variation means that the stability of nonsense transcripts may significantly differ in the relevant tissues from the stability determined in commonly used cell lines.

## Methods

### Mice, cells and tissues

Heterozygous mice carrying the deleted *Men1 *allele (*Men1*^+/Δ^) are from a mixed 129/Sv and C57BL/6 background [17]. Mouse embryonic fibroblasts (MEF) were isolated from littermates of E12.5 embryos derived from intercrosses of *Men1*^+/Δ ^mice [17] and immortalized according to 3T3 protocol published previously [38]. Tissues (stomach, liver, heart, spleen, lung, mammary gland, kidney, ovary, testis, brain, intestine, thymus and adrenal) were harvested from 4 two month-old *Men1*^+/Δ ^mice (2 male and 2 female), briefly washed in phosphate-buffered saline (PBS), quickly frozen in liquid nitrogen and transferred for long term storage at -80°C.

All animal experiments were conducted in accordance with the standards of human animal care and were approved by the International Agency for Research on Cancer's Animal Care and Use Committee.

### Cell culture

MEF cells were maintained in Dulbecco's modified Eagle medium (Invitrogen, Cergy Pontoise, France) supplemented with 10% foetal calf serum, 100 units/ml penicillin, 100 μg/ml streptomycin, 2 mM L-glutamine (VWR, Fontenay sous Bois, France) and 100 μM β-mercaptoethanol in a 5% CO2 incubator at 37°C.

For NMD (translation) inhibition, cells were treated with a fresh puromycin solution (Sigma-Aldrich, St Quentin Fallavier, France) at the final concentration of 100 μg/ml of culture for 6 hours and harvested, washed once with PBS, and subsequently used for total RNA isolation.

### RNA extraction and reverse transcription

Frozen tissues were homogenized in liquid nitrogen using a CRYOPIX^® ^grinder (Alphelys, Plaisir, France). Total RNA was isolated from 5 × 10^6 ^MEF cells or from ~30 mg of tissue powder using the NucleoSpin RNA II kit (Macherey-Nagel, Hoerdt, France), according to the manufacturer's instructions.

RNA concentrations were quantified with a *Bio*photometer (Eppendorf, Le Pecq, France) and integrity of RNA was checked by 1% agarose gel electrophoresis. Protein contamination was monitored by A260/A280 ratio. All samples had a ratio between 1.7 and 2.

A constant amount of 500 ng of total RNA was reverse transcribed in a total volume of 20 μl using 20 pmol of oligo(dT) primers (Promega, Charbonnières, France) and 50 units of Expand Reverse Transcriptase (Roche, Meylan, France) according to the manufacturer's instructions. All tissues of each mouse were reverse transcribed at the same time, and all investigated samples have been reverse transcribed using the same batch of oligo(dT) and enzyme.

### Real-time PCR

PCR amplification and analysis were achieved using a LightCycler 2.0 instrument (Roche) and software version 4.0, respectively (Roche). The specific primers for quantitative real-time PCR were designed using publicly available sequences from the Nucleotide Sequence Database (see Additional file 1). To amplify wild-type *Men1 *transcripts, we used a forward primer complementary to a sequence localised in exon 3 (absent in the mutant *Men1 *transcript species) and a reverse primer complementary to a sequence localised at the exon 3/exon 4 junction. To amplify mutant *Men1 *transcripts, we used a forward primer complementary to a sequence localised in exon 2 and a reverse primer complementary to a sequence localised at the exon 2/exon 4 junction. The primers designed to amplify the transcripts of the genes encoding NMD factors, splicing factors, or the control genes also span at least one exon-exon junction (except for *Upf3b *and *Hprt1*). All primers were synthesized by MWG Biotech AG. Master-mix for each PCR run was prepared in a total volume of 10 μl with 2.5 or 5 mM MgCl_2 _(see Additional file 2), 0.5 μM of each primer, 1 μl LightCycler Fast Start DNA Master SYBER Green I mix, and 2 μl of cDNA. All templates were amplified using the following protocol: after 10 min of denaturation at 95°C, samples were subjected to 40 cycles of denaturation for 15 s at 95°C, annealing for 5 s at 64°C or 66°C (see Additional file 1), and elongation for 10 s at 72°C. A melting curve was performed at the end of the 40 cycles by heating the samples at a rate of 0.2°C/s starting at 70°C up to 97°C with continuous measurement of fluorescence. The specificity of the real time RT-PCR reaction performed to amplify *Men1 *wild-type or mutant transcript species was controlled using RNA extracted from *Men1*^+/+ ^and *Men1*^-/- ^MEF cells.

Each sample was analysed by at least three PCR reactions. Absolute quantification of RNA copy number was determined using a standard curve that is generated with serial dilutions of a PCR product with a known concentration (as measured with the *Bio*photometer).

### NMD efficiency and transcript levels assessment

NMD efficiency is expressed as the ratio of the number of mutant versus wild-type *Men1 *RNA copies in each tissue. To assess the level of expression of the 11 genes encoding proteins involved in NMD and of the wild-type *Men1 *allele, in each of the 13 tissues, the number of RNA copies was normalized with the average number of RNA copies for the *Hprt1 *and *β*-*actin *genes. Copy numbers of the transcripts of these two genes are shown in Additional file 1.

### Statistical analysis

To test the significance of the differences between tissues, transcript measurements being nested within tissue and tissues being nested within mouse, data were modelled using a hierarchical mixed model [39]. The "tissue" factor was studied as a fixed effect and the "mouse" factor embedded within the "tissue" factor as a random effect in order to take into account the within-mouse correlation. Estimations were done using the maximum likelihood method using the nlme library of the software R version 2.2.1 [40]. The statistical significance level was 0.05.

## Authors' contributions

ABZ carried out the bench work. SF carried out the dissection of the mice. DM performed the statistical analysis. MB participated in the bench work. CXZ participated in the design and coordination of the study and helped to draft the manuscript. SM conceived of and designed the study, and drafted the manuscript. All authors read and approved the final manuscript.

## Supplementary Material

Additional File 1 **Primers and parameters used for the quantitative RT-PCR analysis**. Click here for file

Additional File 2 **Transcript levels of the *Hprt1 *and *β*-*actin *genes in 13 murine tissues**. Click here for file
