# Supplementary material for: Comparison of nonsense-mediated mRNA decay efficiency in various murine tissues
Source: BMC Genet. 2008 Dec 5;9:83. doi: 10.1186/1471-2156-9-83 (PMC2607305; doi:10.1186/1471-2156-9-83)
Supplement: Additional File 1 — Primers and parameters used for the quantitative RT-PCR analysis. [file 1471-2156-9-83-S1.pdf]

Additional Table 1 - Primers and parameters used for the quantitative RT-PCR analysis.

Gene	GeneBank accession number	Primer sequence	Localization	PCR fragment size (nt)	$MgCl_2$ concentration (mM)	Annealing temperature (°C)
Wild-type *Men1*	NM_008583	F: atc atg ctt ggg tgg tgt tt	Exon 3	128	2.5	66
		R: gta cag cca gct ccg ctc a	Junction 3-4			
Mutant *Men1*		F: cgg atg tca tat gga aca gc	Exon 2	96	5	66
		R: cag gta cag cca gct ctg tg	Junction 2-4			
Upf1	NM_030680	F: gcc tgc agt tac tgt gga atc	Junction 3-4	188	5	66
		R: tag cac tcc agc acg gtc t	Exon 5			
Upf2	NM_001081132	F: tgc taa gac caa aga tca aac tc	Exon 15	248	5	64
		R: ctc ctc ctc aga acc ctc tt	Junction 16-17			
Upf3a	NM_025924	F: gcg gac ctc agc ctt tat c	Junction 2-3	172	5	64
		R: ttt tgg caa tct tct gga atg	Exon 4			
Upf3b	NM_026573	F: cag gga ccg att tga tgg	Exon 3	187	5	66
		R: ctc att gtc tgt ggc ata act ctc	Exon 5			
Smg1 (RIKEN)	NM_001031814	F: gac cag cct aca atc cat cct	Exon 7	208	5	66
		R: caa act ctg caa cca ccc a	Junction 8-9			
Casc3	NM_138660	F: acg gga act tcg agg tgt g	Junction 7- 8	207	2.5	66
		R: ccc tgg aac tgc aat gga t	Junction 9 - 10			
y14 (Rbm8a)	NM_025875	F: atg agg atg ggg acg aaa g	Junction 1-2	169	2.5	66
		R: aga atc caa cct tca aca gag c	Junction 3-4			
Mago	NM_010760	F: tca gga aag agg ctt atg tgc	Junction 2-3	211	5	66
		R: gga tcc ttg gac tgg ttg ac	Junction 4-5			
Eif4a3	NM_138669	F: gag aaa ccc aag ctt tga tcc	Exon 4	204	2.5	66
		R: ggc gga tca tat caa aga cg	Junction 5-6			
Rnps1	NM_009070	F: cca gga atg tga cca agg at	Junction 6-7	185	5	66
		R: gcc atc aat ttg tcc tcc a	Junction 7-8			
Srrm1	NM_016799	F: gaa gaa cct ccg gta aag tga c	Junction 9-10	197	5	66
		R: gga gcc aga gtc aga tga aga	Junction 11-12			
β-actin	NM_007393	F: agg gtg tga tgg tgg gaa	Junction 2-3	258	2.5	64
		R: agg tct caa aca tga tct ggg	Junction 3-4			
Hprt1	NM_013556	F: tgt tgt tgg ata tgc cct tg	Exon 8	111	5	64
		R: aac ttg cgc tca tct tag gc	Exon 9			
