# Supplementary material for: Comparison of nonsense-mediated mRNA decay efficiency in various murine tissues
Source: BMC Genet. 2008 Dec 5;9:83. doi: 10.1186/1471-2156-9-83 (PMC2607305; doi:10.1186/1471-2156-9-83)
Supplement: Additional File 2 — Transcript levels of the Hprt1 and β-actin genes in 13 murine tissues. [file 1471-2156-9-83-S2.pdf]

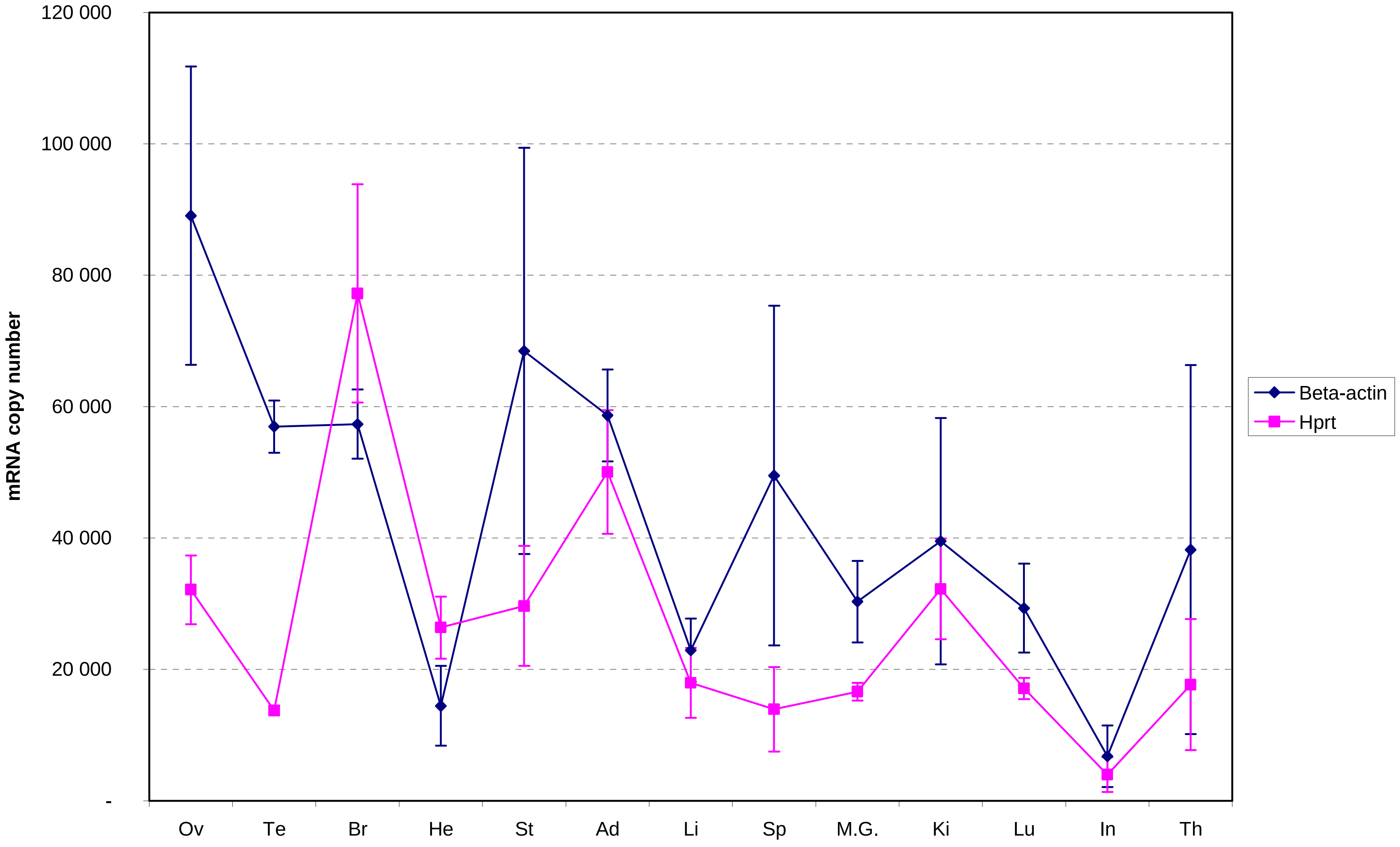

Additional Figure 1 - Transcript levels of the *Hprt1* and *β-actin* genes in 13 murine tissues

Transcript levels (copy number per 50 pg of RNA extracted from each of the mentioned tissues) were measured by quantitative RT-PCR using the same samples as previously. Each bar on the graph represents the mean value of at least 3 independent measurements (± standard deviation) in four samples of the same tissue (two samples only were used for testis and ovary). Ov: ovary; Te: testis; Br: brain; He; heart; St; stomach; Ad: adrenal gland; Li: liver; Sp: spleen; M.G.: mammary gland; Ki: kidney; Lu: lung; In: intestine; Th: thymus.
